# Effects of Online Psychological Crisis Intervention for Frontline Nurses in COVID-19 Pandemic

**DOI:** 10.3389/fpsyt.2022.937573

**Published:** 2022-07-12

**Authors:** Chunyan He, Shuying Chang, Ying Lu, Hongmei Zhang, Haining Zhou, Yunfei Guo, Bu-Lang Gao

**Affiliations:** Henan Provincial People's Hospital, Zhengzhou, China

**Keywords:** COVID-19, psychological crisis, crisis intervention, online, nurses

## Abstract

**Objective:**

The psychological problems of frontline nurses in COVID-19 prevention and control are very prominent, and targeted intervention is needed to alleviate them. This study was to assess the impact of online intervention programs on psychological crisis of anxiety, depression levels and physical symptoms among frontline nurses fighting the COVID-19 pandemic.

**Methods:**

A three-stage online psychological crisis intervention program was established. The General Anxiety 7 (GAD-7) assessment, Patient Health Questionnaire-9 (PHQ-9), and the Self-rating Somatic Symptom Scale (SSS) were used to evaluate the effect of intervention on the day before entering isolation wards (Time 1), the first day after leaving the isolation ward (Time 2), and at the end of the intervention (Time 3).

**Results:**

Sixty-two nurses completed the study, including 59 female (95.2%) and three male nurses (4.8%) with an age range of 23–49 (mean 33.37 ± 6.01). A significant (*P* < 0.01) difference existed in the scores of GAD-7, PHQ-9, and SSS at different intervention periods. The GAD-7 score was significantly (*P* < 0.001) lower at the end of quarantine period (time 3) than that before entering the isolation wards (time 1) or after leaving the isolation wards (time 2), the PHQ-9 score was significantly (*P* = 0.016) lower at the end of quarantine period (time 3) than that after leaving the isolation wards (time 2), and the SSS score was significantly (*P* < 0.001) lower at the end of quarantine period (time 3) than that before entering the isolation wards (time 1) or after leaving the isolation wards (time 2).

**Conclusion:**

The three-stage online intervention program based on the psychological crisis can be effective in reducing negative emotions and somatic symptoms and improving the mental health of frontline nurses in prevention and control of the COVID-19 epidemic. It may provide an empirical basis for psychological crisis intervention of frontline medical staff when facing public health emergencies.

## Introduction

In recent years, public health incidents caused by infectious diseases have occurred frequently around the world, posing a serious threat to people's health and safety (1). Clinical frontline medical staff not only need to complete huge medical treatment tasks, but also face higher infection risk, producing a great psychological burden, especially for women and frontline nurses, who are more likely to have negative psychological problems (2). A number of surveys show that 37.97% of frontline nursing workers have anxiety symptoms to varying degrees and that 32.23% have a high degree of psychological pressure (3–5). The mental health of frontline nurses in epidemic prevention and control is not optimistic (3–5).

Psychological aid is an important approach in helping people have experienced disaster and public health emergencies. In China, psychological crisis interventions play an essential role in terms of preventing and dealing with the psychological problems (such as distress, depression and anxiety) among sufferers and medical staff like doctors and nurses as well. These psychological interventions have been applied in severe acute respiratory syndrome (SARS) outbreak (6) and earthquake disasters (7). The results of psychological crisis intervention were remarkable in reducing depression, anxiety and post-traumatic stress disorder (PTSD) symptoms for victims of public health events or disaster emergencies (6, 8). What's more, psychological crisis intervention can relieve insomnia and improve the quality of life as well. Thus, it is imperative to apply a psychological crisis intervention model to deal with the mental health issues and psychological stresses in people who fight the COVID-19 pandemic.

The National Health Commission issued a notice on emergency psychological crisis intervention in the outbreak of pneumonia infected by novel coronavirus, clearly pointing out that it is necessary to give full play to the existing psychological assistance hotline and a variety of online communication means to provide online psychological assistance services for frontline medical staff including nurses (9). At present, most of the studies just reported the negative emotions of frontline nurses without designing an online intervention model. This study drew on the psychological crisis intervention model of medical staff during SARS and divided the psychological crisis into three states: pre-crisis equilibrium state, crisis generation and post-crisis equilibrium state. This was to accurately understand and analyze the psychological crisis response and characteristics of frontline medical staff in epidemic events and to achieve the best effect of psychological intervention. In the current study, a three-stage online psychological crisis intervention program was established to evaluate and relieve the mental stresses in frontline nurses in epidemic prevention and control at different stages of public health emergencies.

## Materials and Methods

### Participants

This study was approved by the ethics committee of our hospital, and all participants had signed the informed consent to participate. One-hundred and twenty nurses who worked in the isolation wards for the COVID-19 pandemic in our hospital from January to February 2020 were enrolled to test the effect of the three-stage psychological intervention program. A total of 62 nurses completed the intervention program and were the subjects of this study. The inclusion criteria were nurses who worked in the isolation wards in our hospital and provided written informed consent to participate. The exclusion criteria were nurses with apparent psychological problems including anxiety, depression or somatization symptoms. They would be excluded if the scores of anxiety, depression and somatization symptoms were moderate or above. If a single scale was moderate or above, the psychiatrist would make professional judgment to determine whether they were suitable for exclusion. Otherwise, they would continue the one-on-one psychological counseling and follow-up in later stages.

### Psychological Crisis Intervention

**Figure 1:**
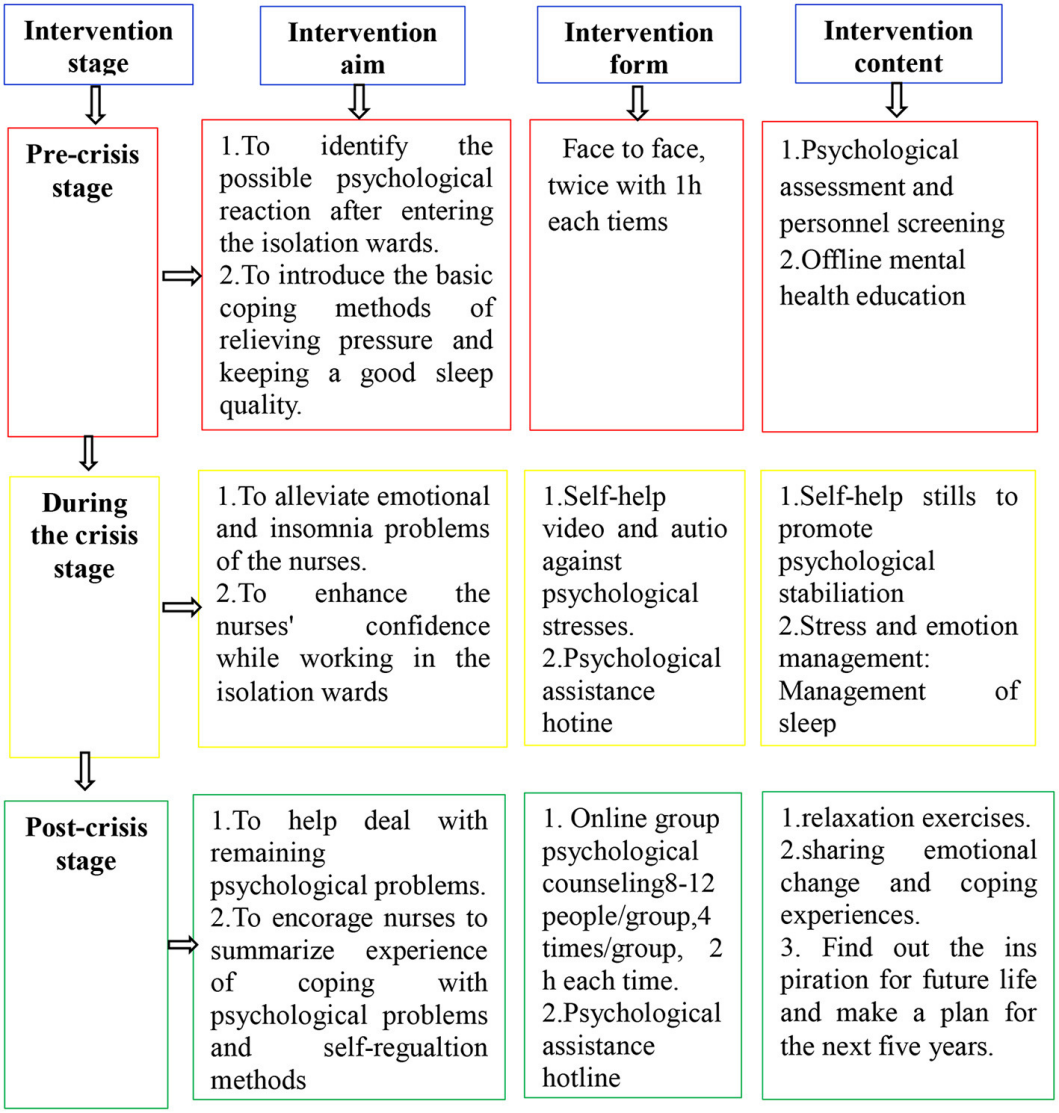
A three-stage psychological crisis intervention model.

A three-stage interventional model for assessing psychological crisis in the COVID-19 pandemic was developed based on the model supposed by Wang (6). This three-stage model divided the psychological crisis intervention into three stages: pre-crisis, mid-crisis, and post-crisis (Figure 1). The focus and methods of intervention varied at different stages. Before the crisis, the psychological resistance ability was enhanced through mental health education and life guidance in medical staff when confronting the crisis. During the crisis, specific target individual or group counseling was provided according to the response of medical staff to the crisis. After the crisis, medical staff were trained to learn lessons from the crisis and develop effective self-regulation methods to maintain mental health.

At the pre-crisis stage of 1–3 days before entering the isolation wards of COVID-19, psychological training was performed for the frontline nurses, and the objective of intervention was to teach nurses how to identify possible psychological reactions to the stress caused by COVID-19, master basic coping methods, and increase their psychological preparation for the crisis. The main contents included methods of identifying and coping with common psychological problems and self-help techniques of relaxation and anxiety alleviation. While working in the isolation wards during the crisis, the purpose was to alleviate emotional and agrypnia problems of the nurses. While working in the isolation wards, nurses received information through the Wechat online connection. On the 2nd, 4th, 7th and 11th days after entering the isolation wards, psychotherapists sent video and audio-based files including self-help techniques as well as official and positive text-based pandemic information to instruct the psychological self-help. If an individual needed psychological assistance, he or she could contact the psychotherapist directly by phone call or Wechat connection. At the post-crisis stage, the nurses left the isolation wards, and a 14-day quarantined medical observation period was required for any medical staff at the designated locations. At this stage, the intervention started on the third day after leaving the isolation wards and performed four times with an interval of 2 days. The objective was to help deal with the remaining psychological problems, draw lessons, perform effective self-regulation, and restore to the pre-crisis psychological level.

### Evaluation Tools

The General Anxiety 7 Assessment (GAD-7), Patient Health Questionnaire-9 (PHQ-9), and Self-rating Somatic Symptom Scale (SSS) were used to evaluate the mental health of the nurses. The GAD-7 assessment was firstly developed by Spitzer (10) for screening subjects' anxiety and severity, and its Cronbach's alpha coefficient and test-retest reliability coefficient are 0.898 and 0.856, respectively, including 7 questions of assessment with 4 options for each one: not at all, a few days, more than half of the days, and almost every day. Individual's choices correspond to 0, 1, 2, and 3 points respectively, with a total score ranging 0–21 points. A GAD-7 score of ≤ 4 stands for no anxiety, 5–9 for mild anxiety, 10–14 for moderate anxiety, and ≥15 for severe anxiety (11). The GAD-7 scale's Cronbach' alpha coefficient was 0.921 in our study. The PHQ-9 is a self-assessment tool for depression developed by Spitzer based on the American Diagnostic and Statistical Manual of Mental Disorders (DSM-IV) to screen subjects for depression (12). The Cronbach's alpha coefficient and test-retest reliability coefficient of PHQ-9 are 0.833 and 0.934, respectively, including 9 questions with four options for each: not at all, several days, more than half of the days, and almost every day. Individuals' choices will be corresponded to 0, 1, 2, and 3 points, respectively, with a total score ranging 0–27 points. A PHQ-9 score of ≤ 4 stands for no anxiety, 5–9 for mild anxiety, 10–14 for moderate anxiety, 15–19 for mid to severe anxiety, and ≥20 for severe anxiety (13). In our study, the PHQ-9 scale's Cronbach's alpha coefficient was 0.868. The SSS was compiled by Zhuang et al. (14), and its Cronbach's alpha coefficient and test-retest reliability coefficient are 0.89 and 0.96, respectively, including 20 questions in total, with 9 questions for somatization, 5 for anxiety, 4 for depression, and 2 for anxiety and depression. An SSS score <30 stands for normal, 30–39 for mild, 40–59 for moderate, and ≥60 for severe psychological stress. In our study, the SSS scale's Cronbach's alpha coefficient was 0.952.

### Statistical Analysis

The data were analyzed with the SPSS 25.0 software (IBM, Chicago, IL, USA). Measurement data were presented as mean ± standard deviation and tested with the student *t* test. Enumeration data were presented as number and percentages and tested with the Chi square test or Friedman's Rank Test of K related samples. The statistically significant *P*-value was set at <0.05.

## Results

A total of 62 nurses completed the study, including 59 female (95.2%) and three male nurses (4.8%) with an age range of 23–49 (mean 33.37 ± 6.01) (Table 1). The median year of working was 10 (interquartile range 7.75–16), and nurses with a bachelor's degree accounted for 98.4% (*n* = 57). Nurses in charge accounted for 67.7% (*n* = 42) while head nurses 16.1% (*n* = 10).

**Table 1:** Basic data of study subjects (*n* = 62).

**Variables**	**Data (*n*, %)**
Male	3 (4.8%)
Female	59 (95.2%)
**Age**	
≤ 30 years	17(27.4%)
31–35 years	24 (38.7%)
36–40 years	14 (22.6%)
≥41 years	7 (11.3%)
**Years of working**	
≤ 5 years	12 (19.4%)
6–10 years	20 (32.3%)
11–15 years	12 (19.3%)
16–20 years	12 (19.3%)
≥20 years	6 (9.7%)
**Education**	
≤ High-school degree	5 (8.1%)
Bachelor's degree	56 (90.3%)
Master's degree	1 (1.6%)
**Title**	
Nurse	17 (27.4%)
Nurse in charge	42 (67.7%)
Deputy chief nurse	3 (4.8%)
**Position**	
Nurse	52 (83.9%)
Head nurse	10 (16.1%)

A significant (*P* < 0.01) difference existed in the scores of GAD-7, PHQ-9, and SSS at different intervention periods (Table 2). The GAD-7 score was significantly (*P* < 0.001) lower at the end of 14-day quarantine period (time 3) than that before entering the isolation wards (time 1) or on the first day after leaving the isolation wards (time 2), the PHQ-9 score was significantly (*P* = 0.016) lower at the end of 14-day quarantine period (time 3) than that on the first day after leaving the isolation wards (time 2), and the SSS score was significantly (*P* < 0.001) lower at the end of 14-day quarantine period (time 3) than that before entering the isolation wards (time 1) or on the first day after leaving the isolation wards (time 2).

**Table 2:** Comparison of scores of GAD-7, PHQ-9 and SSS at three stages (*n* = 62).

**Variables**	**GAD-7**		**PHQ-9**		**SSS**	
	**Median (*P25, P75*)**	**Score range**	**Median (*P25, P75)***	**Score range**	**Median (*P25, P75)***	**Score range**
Time 1	2.00 (0, 4.00)	0–10	2.50 (0, 5.00)	0–9	24.00 (22.00, 29.00)	20–46
Time 2	1.00 (0, 4.00)	0–14	3.00 (0, 7.00)	0–16	25.00 (22.00, 36.25)	20–67
Time 3	0.00 (0, 2.00)	0–7	2.00 (0, 4.50)	0–9	22.00 (21.00, 29.00)	20–44
Statistic	31.040	10.296	90.818
*P-value*	<0.001^a^	0.006 ^b^	<0.001^c^

*Time 1: 1–3 days before entering the isolation wards of COVID-19; Time 2: the first day after leaving the isolation wards; Time 3: at the end of 14-day quarantined period. a: comparison between two groups: P (Time 3-Time 1) <0.001; P (Time 3-Time 2) <0.001; P (Time 2-Time 1) = 1.000. b: comparison between two groups: P (Time 3-Time 1) = 1.000; P (Time 3-Time 2) = 0.016; P (Time 2-Time 1) = 0.161. c: comparison between two groups: P (Time 3-Time 1) <0.001; P (Time 3-Time 2) <0.001; P (Time 2-Time 1) <0.001*.

## Discussion

The sudden outbreak and highly infectiousness of the coronavirus pose serious psychological impacts on medical staff (15). The three-stage online intervention program of psychological crisis constructed in this study aimed at solving the common psychological problems of the frontline personnel in the isolated wards with anxiety, depression, and somatic symptoms. As for the current study, female frontline nurses were the main force fighting the COVID-19 pandemic. There was no overall change in anxiety or depression after the second stage of intervention (*P* < 0.05), whereas somatic symptom level increased significantly (*P* < 0.05) but was still within the normal range. After the third intervention, the level of somatic symptoms, anxiety, and depression of nurses in the quarantined medical observation period was significantly lower than that in the working isolation wards period (*P* < 0.05).

The psychological crisis intervention could be useful among nurses with low psychological burden. In the pre-crisis stage, the median depression level was 2.5 points, and the third quartile was 5, which was lower than the average score of people in the center of pandemic in 2019 (4.57 ± 5.75), indicating less psychological stress (16). Although the depression level of some nurses increased during working in the isolation wards period, the depression level of nurses did not change significantly, indicating that the three-stage intervention mode has played a role in the intervention process. Zhang et al. (17) pointed out in their study that frontline medical staff with moderate or greater anxiety and depression showed significant improvement after psychological crisis intervention, whereas medical staff with psychological burden below the moderate level did not show significant decrease in the anxiety level after consultation intervention. However, our study showed that the psychological crisis intervention had a significant relief effect on people with low psychological burden, indicating that the three-stage psychological crisis intervention model is an effective help for frontline nurses with a low anxiety level in relieving the psychological burden.

It seems that somatic symptoms affect nurses even among those with a low psychological burden. Frontline medical staff have a higher risk of being infected with the coronavirus and higher physical and psychological stresses (7). It has been demonstrated that up to 45.9% of residents have different somatic symptoms during the peak period of the epidemic (18) and that 41.0% of frontline medical staff have reported somatic symptoms, such as insomnia (19). In our study, although some nurses had somatic symptoms in isolated wards, they generally returned to a normal psychological level and did not have any somatic symptoms at the end of online intervention (Time 3). Thus, psychological crisis intervention was useful both physically and psychologically.

It is encouraging to note that the intervention was delivered by remote connection approaches to nurses during the crisis stage and post-crisis stage. Due to the highly infectiousness of the COVID-19 pandemic, Solomonov et al. (20) designed a home telephone psychological intervention model during the epidemic period, which significantly reduced the anxiety and depression symptoms of medical staff, indicating that remote psychological intervention can better relieve the psychological burden of medical staff.

An online psychological intervention model was used in the current study, and this online group intervention was adopted because it could achieve a similar effect to an individualized intervention in improving symptoms of acute stress disorder and emotional problems (21). In the online group intervention, group activities were relatively stable without the risk being infected with the coronavirus, and participants could exchange and discuss common experiences and problems, achieving the goal of supporting and encouraging each other. The online group intervention could also promote self-expression, release inner depression, and stimulate inner desire for self-change. Moreover, it could increase social connection and a sense of belonging, reduce loneliness and fear, and help them adapt to normal work and life as soon as possible (22). Compared with individualized interventions, group intervention could help more people at the same time. This advantage was particularly important at the period of the COVID-19 pandemic. The whole program was mainly implemented through a smartphone application, which provided practical evidence for the feasibility of online crisis intervention research. Intervention objectives and emphases at each stage are in line with the concept characteristics of modern Critical Incident Stress Management (CISM) (23).

Different from previous studies, the participants of our study were nurses with a low psychological burden. Using this online psychological intervention program, good effects had been achieved in frontline nurses with improvement of anxiety, depression and somatic symptoms, proving the effectiveness of the three-stage psychological crisis intervention model. It can provide more scientific and accurate guidance for the intervention of psychological stresses at different stages for frontline nurses.

Some limitations also existed in this study, including a single-center study design, Chinese patients enrolled only, no control, and a small cohort of subjects, which may all affect the generalization the outcomes. Nevertheless, our findings suggest that a three-stage online psychological intervention can reduce psychological burden among frontline nurses. Future studies will have to resolve all the above issues for better clinical outcomes in the intervention of psychological stresses in medical staff, nurses in particular, when fighting a public emergency.

In conclusion, the three-stage online intervention program based on the psychological crisis can be effective in reducing negative emotions and somatic symptoms and improving the mental health of frontline nurses in prevention and control of the COVID-19 pandemic. It may provide an empirical basis for psychological crisis intervention of frontline medical staff when facing public health emergencies.

## Data Availability Statement

The original contributions presented in the study are included in the article/supplementary materials, further inquiries can be directed to the corresponding authors.

## Ethics Statement

The studies involving human participants were reviewed and approved by Ethics Committee of Henan Provincial People's Hospital. The patients/participants provided their written informed consent to participate in this study.

## Author Contributions

HZho participated in interpretation of the data and substantively revised the work. YG proposed amendments and substantively revised the work. All authors contributed to the article and approved the submitted version.

## Funding

This study was supported by Joint Construction Project of Henan Medical Science and Technology Research Plan (LHGJ20210010) and Henan Health Commission for Management of Psychological Pain of Gynecological Cancer Patients (2021-2023).

## Conflict of Interest

The authors declare that the research was conducted in the absence of any commercial or financial relationships that could be construed as a potential conflict of interest.

## Publisher's Note

All claims expressed in this article are solely those of the authors and do not necessarily represent those of their affiliated organizations, or those of the publisher, the editors and the reviewers. Any product that may be evaluated in this article, or claim that may be made by its manufacturer, is not guaranteed or endorsed by the publisher.
